# Cholelithiasis and choledocholithiasis in children; risk factors for development

**DOI:** 10.1371/journal.pone.0196475

**Published:** 2018-05-15

**Authors:** Barbora Frybova, Jiri Drabek, Jindra Lochmannova, Ladislav Douda, Stepan Hlava, Daniela Zemkova, Vladimir Mixa, Martin Kyncl, Lubos Zeman, Michal Rygl, Radan Keil

**Affiliations:** 1 Department of Pediatric Surgery, Charles University in Prague, 2^nd^ Faculty of Medicine, University Hospital Motol in Prague, Prague, Czech Republic; 2 Department of Internal Medicine, Charles University in Prague, 2^nd^ Faculty of Medicine, University Hospital Motol in Prague, Prague, Czech Republic; 3 2^nd^ Department of Internal Medicine–Gastroenterology, Charles University in Prague, Faculty of Medicine in Hradec Kralove, University Hospital Hradec Kralove, Hradec Kralove, Czech Republic; 4 Department of Pediatrics, Charles University in Prague, 2^nd^ Faculty of Medicine, University Hospital Motol in Prague, Prague, Czech Republic; 5 Department of Anaesthesiology and Intensive Care Medicine, Charles University in Prague, 2^nd^ Faculty of Medicine, University Hospital in Motol in Prague, Prague, Czech Republic; 6 Department of Radiology, Charles University in Prague, 2^nd^ Faculty of Medicine, University Hospital Motol in Prague, Prague, Czech Republic; Medizinische Fakultat der RWTH Aachen, GERMANY

## Abstract

**Purpose:**

To compare anthropometric data (body mass index [BMI]) in patients without lithiasis to patients with symptomatic simple cholelithiasis or choledocholithiasis.

**Methods:**

We retrospectively reviewed data from 147 patients undergoing laparoscopic cholecystectomy between 2001–2015. Complete growth data from 98 patients was compared with anthropometric data from the population of the Czech Republic and a control group (BMI of 100 consecutive patients without biliary stones in abdominal ultrasound who were admitted to a surgical department for suspected appendicitis).

**Results:**

The BMI of 75 children with simple cholelithiasis and 23 with choledocholithiasis was compared to the standard Czech pediatric population and to the control group. The median age (simple cholelithiasis and choledocholithiasis) was 16 years, and 35 patients (24%) had a family history of gallstones. Types of lithiasis included multiple (n = 120), solitary (n = 11), and sludge (n = 10). Five cases had polyps and one had gallbladder dysplasia. Patients with simple cholelithiasis had significantly higher BMI compared to the control group without cholelithiasis (p<0.0001) and the standard Czech population (p = 0.03). Patients with choledocholithiasis had a mean BMI significantly higher than that of the general population (p = 0.001) and the control group (p = 0.0001). Patients with choledocholithiasis had significantly higher BMI than those with simple cholelithiasis (p = 0.03).

**Conclusion:**

Patients with cholelithiasis had significantly higher BMI than the general population, and patients with choledocholithiasis had significantly higher BMI than patients with simple lithiasis. Elevated BMI is a risk factor for developing choledocholithiasis. ERCP and early laparoscopic cholecystectomy in patients with choledocholithiasis offer equivalent outcomes in patients with simple cholelithiasis.

## Introduction

Cholelithiasis in children is a rare disease with a prevalence between 0.13 and 0.22%[1]. The main risk factor for cholelithiasis is most likely obesity, and the increased incidence of childhood obesity is becoming alarming [2]. Cholelithiasis is also detected more frequently than in previous years because of the increased use of ultrasound [3].

Nowadays, we focus our attention not only on the rising number of diagnosed cases of infantile cholelithiasis and its operative treatment, but also on the type of cholelithiasis, its possible complications, and the incidence of obesity, specifically the elevation of body mass index (BMI). Therefore, we evaluated anthropometric data from patients with simple cholelithiasis and those with choledocholithiasis and compared them with the general population. We also performed an analysis of long-term results after laparoscopic cholecystectomy due to simple cholelithiasis and choledocholithiasis after an endoscopic retrograde cholangio-pancreatography (ERCP).

## Materials and methods

All patients with cholelithiasis who were operated on via a laparoscopic approach at our Department of Pediatric Surgery between 2001–2015 were included in a retrospective single center analysis. The height and weight were measured in each patient on devices of a standard calibration of known accuracy upon their admission to our department by experienced nurses. All our operated patients had lithiasis confirmed by ultrasound examination. Patients with primary asymptomatic cholelithiasis who had gallstones diagnosed by ultrasound as accidental findings were managed conservatively and treated with ursodeoxycholic acid (dose 10–20 mg/kg/day). Its administration changes the composition of bile and should dissolve gallstones: this conservative therapy was believed to be successful in some patients [4]. However, litholysis using oral bile acids is no longer recommended according to the latest European guidelines published in 2016 due to findings based on adult patients who exhibited low rates of cure and high risk of gallbladder stone recurrence [5]. These asymptomatic patients were followed clinically with examinations approximately twice a year. From the year 2016 administration of bile acids was no longer used.

Cholecystectomy was indicated in patients who underwent at least one episode of biliary colic or had complicated cholelithiasis (cholecystitis or patients after an ERCP due to choledocholithiasis).

Patients with simple cholelithiasis were operated on electively. Patients who had an ERCP due to choledocholithiasis were indicated for early laparoscopic cholecystectomy immediately after their liver enzymes and amylase decreased during the same hospital visit.

The surgical procedure consisted of a four-port laparoscopic cholecystectomy under CO_2_ pneumoperitoneum.

**Fig 1:**
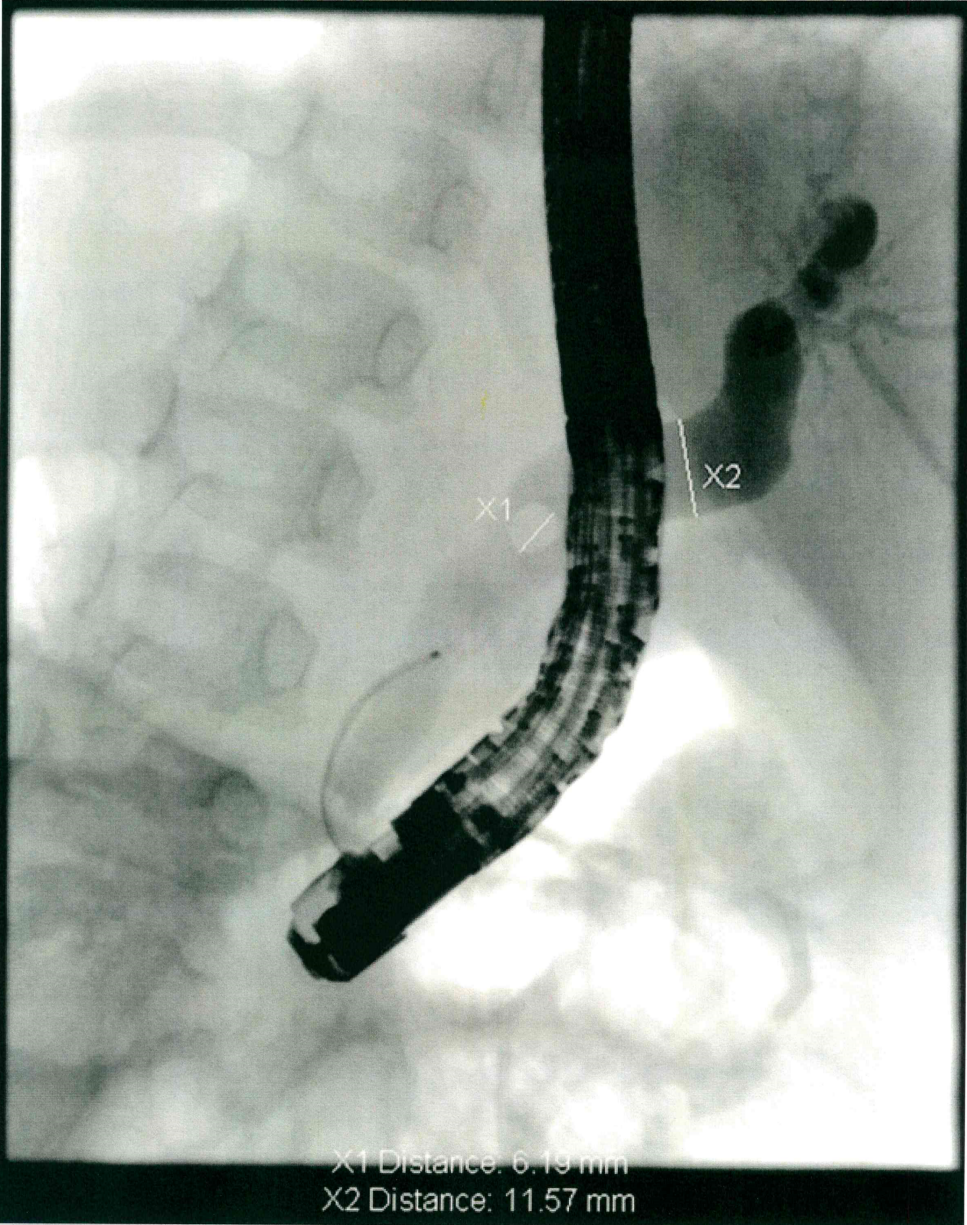
X-ray during ERCP showing choledocholithiasis (X1) and dilatation of the choledochal duct (X2).

ERCP in children was performed by skilled adult endoscopists under general anesthesia and X-ray navigation. A standard adult diagnostic and therapeutic lateroscope (Olympus JF140 R, TJF 160) or special paediatric lateroscope (Olympus PJF 160) were used. Paediatric lateroscope is mandatory in infants younger than 12 months and is preferred for children younger than three years (Fig 1).

The data of the cholelithiasis and choledocholithiasis group were statistically evaluated and compared with the general population by a clinical anthropologist using a statistical Student's T-test and the Mann-Whitney test where appropriate. A p value <0.05 was regarded as significant.

### Study data

Data from the symptomatic cholelithiasis and choledocholithiasis groups were compared to a group of 100 consecutive patients without biliary stones who were admitted to the surgical department for suspected appendicitis and to anthropometric data of the population of the Czech Republic (body height, weight, and BMI by means of SDS (Standard Deviation Score) according to the latest available Czech standards (VI. Nationwide anthropological survey 2001) [6]. The BMI values were related to the percentile growth charts.

The study design was approved by the Ethical Committee of the Second Medical Faculty, Charles University, Prague.

## Results

### Patient characteristics

**Fig 2:**
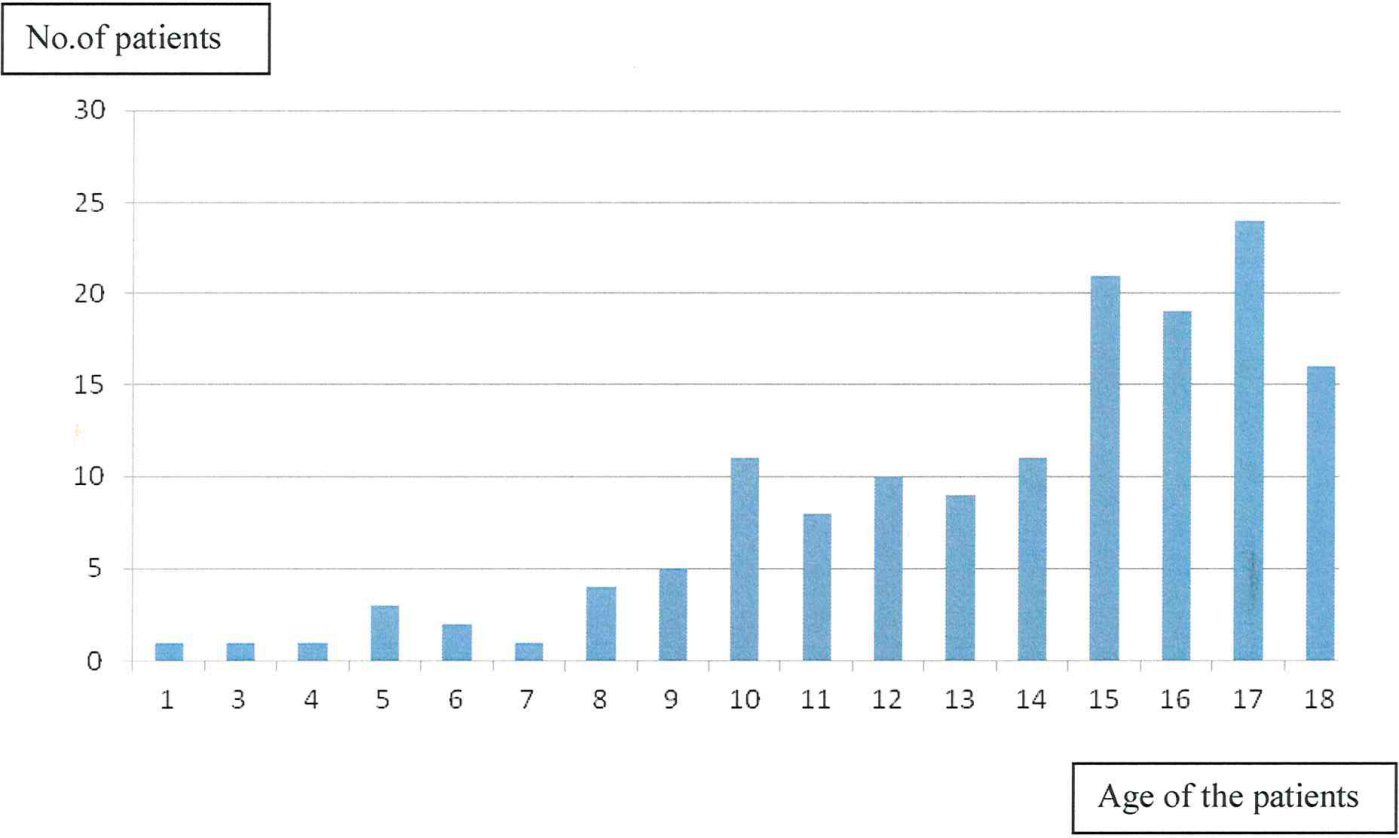
Age range of the operated patients.

Over the 15-year period (2001–2015), 147 patients underwent cholecystectomy via a laparoscopic approach. The median age was 16 years (range, 15 months to 18 years 11 months, Fig 2). There were 103 girls (70%) and 44 boys (30%), and 35 patients (24%) had a family history of gallstones (Table 1).

**Table 1:** Patients characteristics.

Total no.of patients who underwent cholecystectomy	147
Girls	103 girls (70%)
Boys	44 boys (30%)
Mean age of patients	16 years (1–18 years)
Family history of gallstones	35 patients (24%)
Complete data of patients (height, weight)	98 patients
Simple cholelithiasis	75 patients
Choledocholithiasis	23 patients
Control group without cholelithiasis	100 patients

The comorbidities of our study group included hereditary spherocytosis (n = 7), cystic fibrosis (n = 1), scleroderma (n = 1), ganglioneuroblastoma (n = 1), oncological diseases after chemotherapy treatment (n = 5; 1 each of myelodysplastic syndrome, pelvic osteosarcoma, orbital myelosarcoma, nephroblastoma, and acute myeloid leukemia). Further comorbidities were congenital heart disease (n = 3), pancreas divisum (n = 1), Crohn disease (n = 1), Hirschsprung disease (n = 1), epilepsy (n = 2), thyroiditis (n = 2), and celiac disease (n = 1).

Ninety five patients (64.6%) had simple cholelithiasis (i.e., underwent an episode of biliary colic). Fifty two patients (35.4%) had choledocholithiasis; these patients underwent an ERCP due to clinical, laboratory, or ultrasound signs of bile duct obstruction. Fifteen patients (10.2%) had serious acute biliary pancreatitis.

### Anthropometric data

Complete growth data (height, weight, and BMI) were obtained for 98 patients from our study group after laparoscopic cholecystectomy.

The control group consisted of 100 consecutive patients without cholelithiasis admitted to the department of pediatric surgery with suspicion of appendicitis. The median age of this group was 16 years (range 10 to 18 years). There were 57 girls (57%) and 43 boys (43%), with a mean BMI of 18.54 ± 3.1 kg/m^2^ (range 12.6 to 28.0 kg/m^2^).

**Fig 3:**
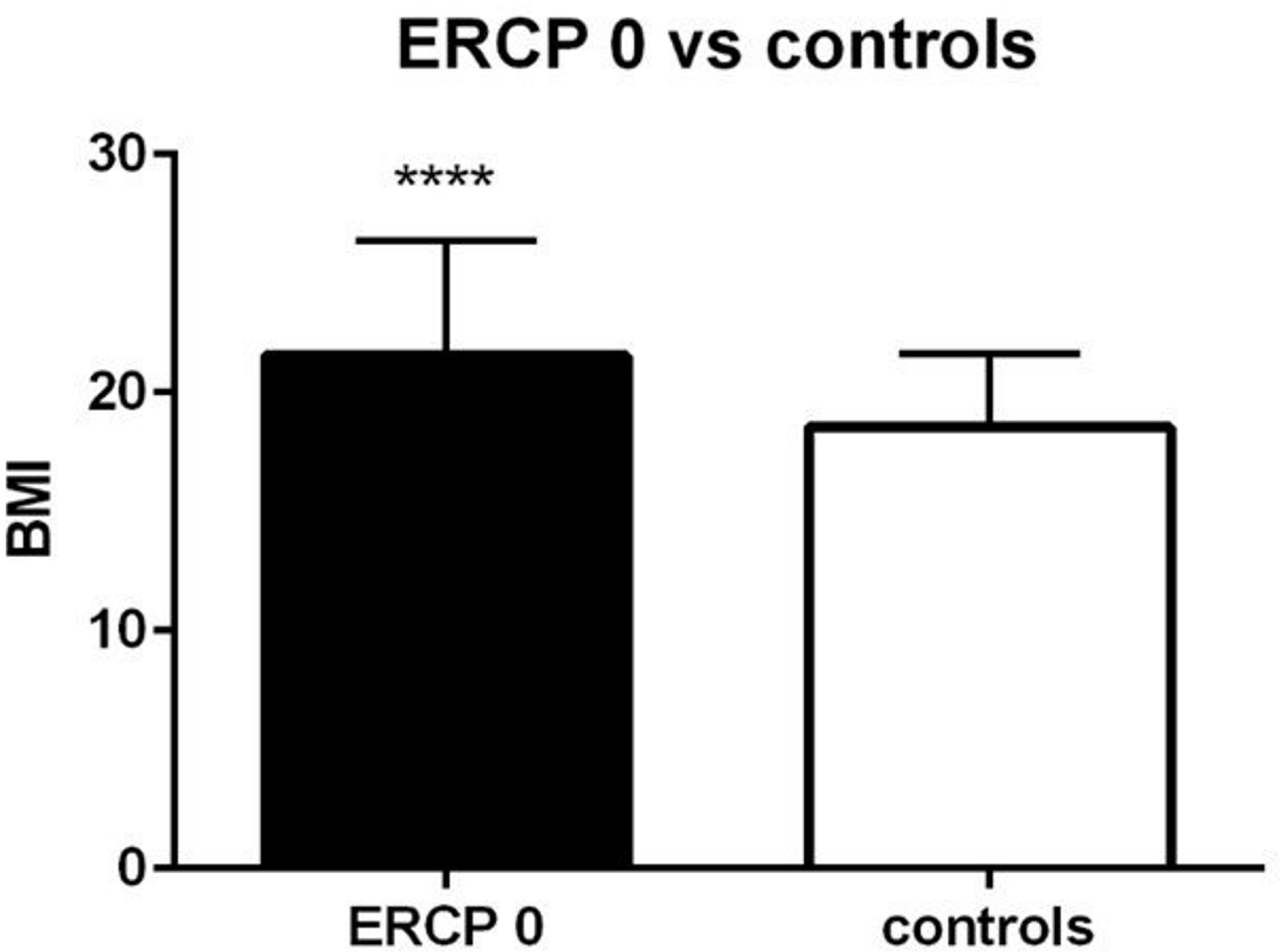
Comparison of the BMI of patients with simple cholelithiasis who did not undergo ERCP (ERCP 0) with that of the control group.

**Fig 4:**
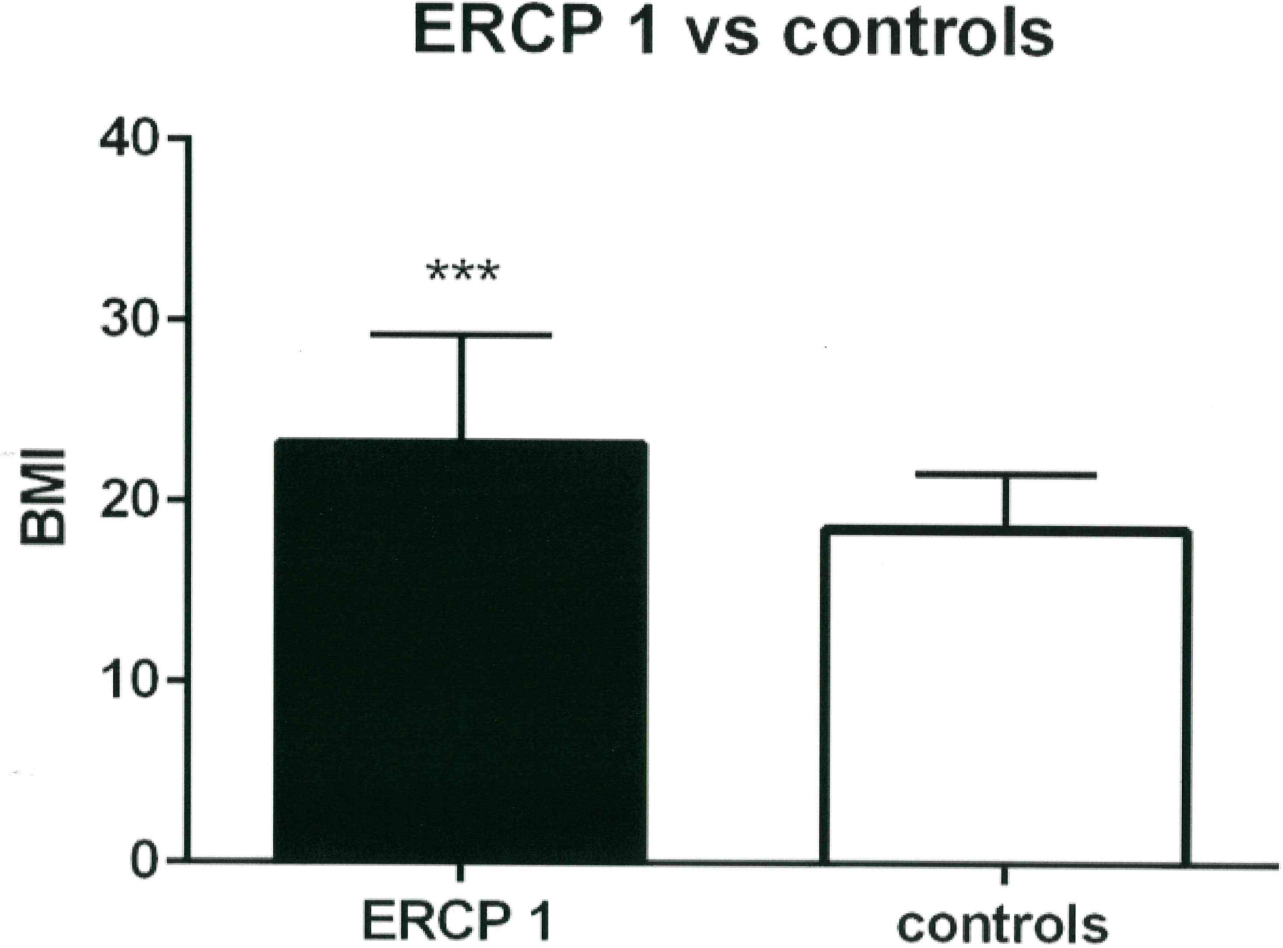
Comparison of the BMI of patients with choledocholithiasis who underwent the ERCP procedure (ERCP 1) with that of the control group.

Patients with simple cholelithiasis (n = 75) had a mean BMI of 21.5 ± 4. 8 kg/m^2^ (range 13.6 to 40.1 kg/m^2^), SDS of 0.43 ± 1.43 kg/m^2^, which was significantly higher compared to the general population (p = 0.03) (Table 2) and to the control group (BMI 18.54 ± 3.1 kg/m^2^, p<0.0001) (Table 3, Fig 3). Patients with choledocholithiasis who underwent an ERCP (n = 23) had a mean BMI significantly higher than that of the general population (BMI 23.3 ± 5.4 kg/m^2^; range 13.9 to 36.8 kg/m^2^; SDS 1.1 ± 1.26 kg/m^2^, p = 0.001) (Table 2) and also higher compared to our control group (BMI 18.54 ± 3.1 kg/m^2^, p = 0.0001) (Table 3, Fig 4). Comparing the simple cholelithiasis and choledocholithiasis (with ERCP) groups, patients with choledocholithiasis had significantly higher mean BMI (p = 0.03). All p values were statistically significant.

**Table 2:** Comparison of patients with cholelithiasis with the anthropometric data of the Czech Republic.

	BMI SDS (kg/m^2^)	*p*
Simple cholelithiasis (n = 75)	0.43 ± 1.43	0.03
Choledocholithiasis, ERCP (n = 23)	1.1 ± 1.26	0.001

BMI (body mass index); ERCP (endoscopic retrograde cholangio-pancreatography); SDS (standard deviation score)

**Table 3:** Comparison of patients with cholelithiasis with those in the control group.

	Mean BMI (kg/m^2^)	*p*
Control group (n = 100)	18.54 ± 3.1	
Simple cholelithiasis (n = 75)	21.5 ± 4.8	<0.0001
Choledocholithiasis, ERCP (n = 23)	23.3 ± 5.4	0.0001

BMI (body mass index); ERCP (endoscopic retrograde cholangio-pancreatography)

### Surgical complications and outcome

The conversion rate into laparotomy was 2% (n = 3), including one conversion due to the presence of multiple adhesions where the surgical team was not able to isolate the cystic duct safely. Another conversion was due to advanced fibrosis of the Callot triangle and the last one because of significant bleeding from the gallbladder bed.

There was no need to perform intraoperative cholangiography. Obesity did not have an affect on operative parameters.

We used Redon drain in 37 patients (25%). The drain was always extracted between the first and second postoperative day. Our use of drains has been steadily decreasing during the years of our study period due to our improving confidence with the laparoscopic technique.

The average length of hospital stay after laparoscopic cholecystectomy was 3.9 days. Mean time to first feeding was 19 hours. Neither the average length of hospital stay nor the mean time to first feeding differed in both the simple cholecystolithiasis and the choledocholithiasis group. The recovery was similar in both groups.

Pathologic examination revealed important chronic cystitis (with a presence of fibrosis, lymphocytes, intraepithelial neutrophils and entrapped epithelial crypts) in 11 cases (7.5%). The types of lithiasis were: multiple (n = 120, 81.6%), solitary (n = 11, 7.5%), and sludge (n = 10, 6.8%). Polyps were found in 5 cases (3.4%), dysplasia of gallbladder in one (0.7%).

Complications occured in 2.7% of patients (n = 4). During the postoperative period we noticed one severe bleeding into the Redon drain (it was a patient who had undergone early cholecystectomy after ERCP due to choledocholithiasis), but we managed to treat it conservatively with blood transfusion. There were two cases of wound dehiscence in our study group (both in patients with simple cholelithiasis) and one girl who had to undergo an ERCP 3 years after laparoscopic cholecystectomy due to the development of choledocholithiasis. The ERCP had not been performed on this girl before.

The postoperative evolution in all cases was favourable, no major morbidity was observed nor did any deaths occur in our group (0% mortality).

We found no differences in outcomes for patients with simple cholelithiasis versus those with choledocholithiasis (course of operation, intra- and postoperative complications, length of hospital stay, family history, or follow-up) after laparoscopic cholecystectomy. Even patients with pancreatitis did not influence the final results.

## Discussion

The association between obesity and metabolic syndrome with cholelithiasis is well described in adults. Moreover, some interesting studies concerning this relationship have been published in the pediatric population as well. Kaechele et al. investigated the prevalence of gallbladder stone disease in a collective of obese children who had undergone ultrasound for detection of gallbladder stone disease. Gallbladder stones were detected in 10 of 493 obese patients in their study (2.0%) [7]. Kobnick et al. published a large population-based cross-sectional study of children in California aged between 10 and 19 years. They identified 766 patients with gallstones from medical records of 510,816 patients. A significantly higher incidence of cholelithiasis was seen in obese children and in girls who used oral contraceptives [8]. Fradin et al. confirmed the relationship by comparing the rising BMI of the pediatric population with the increasing incidence of hospitalization for cholelithiasis in New York in his retrospective case-controled study. The study suggests that obesity is a significant risk factor for hospital admission because of cholelithiasis [9].

Our results show a significantly higher BMI in children with symptomatic cholelithiasis compared to a control group of randomly examined children with sonographically excluded cholelithiasis and to the Czech pediatric population according to the latest available Czech standards (VI. Nationwide anthropological survey 2001) [6]. This result has been predicted in a range of published studies [7,8,9]. Unexpected and yet unpublished findings show a significant correlation between BMI and the risk of choledocholithiasis in children.

Obese children with a mean BMI of 21.5 already have a significantly higher risk of choledocholithiasis compared to children with a normal BMI. However, children suffering from symptomatic cholecystoliatis with a mean BMI of 23 or more find themselves even more at risk for development of choledocholithiasis, showing symptoms at asignificantly higher rate than both the general and obese population.

On behalf of the results of our study we suggest that children with biliary symptoms and cholecystolithiasis as well as unclear ultrasonography of the bile ducts with a BMI of 23 or more should routinely undergo MRCP for exclusion or confirmation ofcholedocholithiasis.

Two possible hypotheses could explain why children with higher BMI are more likely to develop choledocholithiasis rather than simple cholecystolithiasis. The “mechanical” hypothesis could be considered, which postulates that higher body fat content in the abdominal wall causes greater intra-abdominal pressure, resulting in higher direct pressure on the external wall of the gallbladder. The relatively higher pressure in the subhepatal region is thought to cause a direct stimulus that dislodges the concrement from the gallbladder into the extrahepatic biliary tree system, finally causing obstruction of the choledochus, with predictable consequences. This explanation is limited at the point in the relationship between higher BMI and higher intra-abdominal pressure. This relationship is well known in adult patients, but there is no correlation between BMI and intra-abdominal pressure in pediatric patients [10–14]. This finding weakens the “mechanical” hypothesis. Another reason for the higher incidence of choledocholithiasis in obese children should also be considered.

In the “humoral” hypothesis, the reason for a higher risk of choledocholithiasis in relation to higher BMI may be more valid. A diet rich in fat seems to trigger several steps leading to biliary concrement formation and output into the biliary tree system. First, there is a hypersaturation of bile caused by either increased hepatic cholesterol uptake or increased de novo cholesterol synthesis. Second, dysmotility and impaired contraction of the gallbladder is caused by the direct influence of cholesterol at the cellular level onto the plasma membrane of smooth muscle cells in the gallbladder wall [15]. The role of higher levels of cholecystokinin in obese children as a major humoral stimulus for postprandial gallbladder emptying seems to be essential in this matter [16].

From the morphological point of view, the significantly enlarged gastric antral area formed overnight in fasting obese children provides greater gastric capacity and, secondarily, more substrate in the hyperosmotic chyme solution, leading to more intense excretion of cholecystokinin [17]. We feel that the “humoral” hypothesis is more likely to account for the relationship between higher BMI in children and the risk of choledocholithiasis, although the “mechanical” hypothesis may still play a contributing role.

Another point that should be taken into account (and is well described in adults) is the relationship between nonalcoholic fatty liver disease (NAFLD) and cholelithiasis. Prevalence of cholelithiasis is significantly higher in patients with NAFLD [18]. It is expected that both NAFLD and cholelithiasis belong to complications of insulin resistance and metabolic syndromes sharing similar pathogenic paths [19]. NAFLD is the most common liver disease in childhood [20] and, as well as in adults, has a strong correlation to obesity and metabolic syndrome [21]. Consequences of NAFLD and cholelithiasis in childhood are plausible, but should be confirmed by specific studies in the future.

The incidence of obesity in the pediatric population is rising in the Western World. Therefore, the incidence of symptomatic cholelithiasis is predicted to also increase. From our point of view and in accordance with the literature, operational results after cholecystectomy are BMI independent, and there are no differences in outcomes in children with simple cholelithiasis versus choledocholithiasis [22,23].

## Conclusion

The anthropometric data confirmed that patients with cholelithiasis have a higher BMI compared to the normal population. Patients with choledocholithiasis had a significantly higher BMI compared with children with simple cholelithiasis. Children with symptomatic cholecystolitiasis with higher BMI (values close to 23 kg/m^2^ and higher) have significantly higher risk of choledocholithiasis compared to the less obese population. Nevertheless, higher BMI does not influence post-operative outcomes.
